# Supplementary material for: Combining Different Docking Engines and Consensus Strategies to Design and Validate Optimized Virtual Screening Protocols for the SARS-CoV-2 3CL Protease
Source: Molecules. 2021 Feb 4;26(4):797. doi: 10.3390/molecules26040797 (PMC7913849; doi:10.3390/molecules26040797)
Supplement: Supplementary file 1 [file molecules-26-00797-s001.zip › molecules-1018767- Supplementary materials - XML conversion.docx]

Supporting Materials

Combining different docking engines and consensus strategies to design and validate optimized virtual screening protocols for the SARS-CoV-2 3CL protease

Candida Manelfi^1^, Jonas Gossen^2,3^, Silvia Gervasoni^4^, Carmine Talarico^1^, Simone Albani^2,3^, Benjamin Joseph Philipp^2,3^, Francesco Musiani^5^, Giulio Vistoli^4^, Giulia Rossetti^2,6,7^, Andrea Rosario Beccari^1^ and Alessandro Pedretti^4*^

^1^ Dompé Farmaceutici SpA, Via Campo di Pile, 67100, L’Aquila, Italy; carmine.talarico@dompe.com (C.T.); candida.manelfi@dompe.com (C.M.); andrea.beccari@dompe.com (A.R.B.);

^2^ Computational Biomedicine, Institute for Neuroscience and Medicine (INM-9) and Institute for Advanced Simulations (IAS-5), Forschungszentrum Jülich, 52425 Jülich, Germany; s.albani@fz-juelich.de (S.A.); j.gossen@fz-juelich.de (J.G.); g.rossetti@fz-juelich.de (G.R.); benjamin.joseph@rwth-aachen.de (B.J.P.)

^3^ Faculty of Mathematics, Computer Science and Natural Sciences, RWTH Aachen, 52062 Aachen, Germany;

^4^ Dipartimento di Scienze Farmaceutiche, Università degli Studi di Milano, Via Mangiagalli, 25, I-20133 Milano, Italy; silvia.gervasoni@unimi.it (S.G.); alessandro.pedretti@unimi.it (A.P.); giulio.vistoli@unimi.it (G.V.);

^5^ Laboratory of Bioinorganic Chemistry, Department of Pharmacy and Biotechnology, University of Bologna, 40127 Bologna, Italy; francesco.musiani@unibo.it

^6^ Jülich Supercomputing Center (JSC), Forschungszentrum Jülich, 52425 Jülich, Germany

^7^ Department of Hematology, Oncology, Hemostaseology, and Stem Cell Transplantation University Hospital Aachen, RWTH Aachen University, Pauwelsstraße 30, 52074 Aachen, Germany

***** Correspondence: alessandro.pedretti@unimi.it; Tel.: +39 02 50319332 (A.P.).

**Table S1.** Occurrence of the various score values as observed in all the consensus models generated by using the PLANTS docking results.

| **Score** | **without spaces** | **isomeric space** | **binding space** | **both spaces merged** | **both spaces joint** | **relative abundance** |
| --- | --- | --- | --- | --- | --- | --- |
| **Primary** | 3 | 72 | 24 | 66 | 0 | 6% |
| **PLANTS** | 141 | 93 | 101 | 180 | 244 | 26% |
| **Xscore** | 242 | 226 | 248 | 170 | 95 | 34% |
| **VEGA** | 4 | 46 | 65 | 16 | 204 | 12% |
| **MLP** | 0 | 23 | 21 | 65 | 37 | 5% |
| **contacts** | 100 | 86 | 115 | 97 | 89 | 17% |
| **APBS** | 0 | 24 | 0 | 1 | 7 | 1% |
| **Type of score values (only for analyses including space descriptors)** | | | | | | |
| **Mean** | --- | 135 | 131 | 113 | 192 | 24% |
| **Best** | --- | 287 | 293 | 327 | 304 | 50% |
| **Spread** | --- | 148 | 150 | 155 | 180 | 26% |

**Table S2.** Occurrence of the various score values as included in all the consensus models generated by using the LiGen results.

| **Score** | **without spaces** | **isomeric space** | **binding space** | **both spaces merged** | **both spaces joint** | **relative abundance** |
| --- | --- | --- | --- | --- | --- | --- |
| **PH distances** | 462 | 483 | 418 | 422 | 349 | 73% |
| **Primary** | 29 | 3 | 5 | 4 | 16 | 2% |
| **PLANTS** | 6 | 6 | 137 | 152 | 95 | 14% |
| **Xscore** | 23 | 27 | 23 | 13 | 37 | 4% |
| **VEGA** | 1 | 2 | 0 | 0 | 6 | 0.3% |
| **MLP** | 0 | 2 | 0 | 0 | 18 | 0.7% |
| **contacts** | 37 | 64 | 5 | 3 | 68 | 6% |
| **APBS** | 0 | 0 | 0 | 0 | 0 | 0% |
| **Type of score values (only for analyses including space descriptors)** | | | | | | |
| **Mean** | --- | 133 | 117 | 126 | 70 | 19% |
| **Best** | --- | 234 | 349 | 301 | 221 | 47% |
| **Spread** | --- | 220 | 123 | 168 | 298 | 34% |

**Table S3.** Occurrence of the various score values as included in all the generated consensus models based on Fred simulations.

| **Score** | **without spaces** | **isomeric space** | **binding space** | **both spaces merged** | **both spaces joint** | **relative abundance** |
| --- | --- | --- | --- | --- | --- | --- |
| **Primary** | 19 | 8 | 6 | 5 | 4 | 1% |
| **PLANTS** | 309 | 392 | 357 | 315 | 362 | 59% |
| **Xscore** | 141 | 188 | 155 | 196 | 181 | 29% |
| **VEGA** | 11 | 20 | 54 | 54 | 44 | 6% |
| **MLP** | 6 | 0 | 0 | 0 | 0 | 0.2% |
| **contacts** | 18 | 1 | 3 | 3 | 31 | 2% |
| **APBS** | 39 | 0 | 0 | 0 | 22 | 2% |
| **Type of score values (only for analyses including space descriptors)** | | | | | | |
| **Mean** | --- | 168 | 183 | 223 | 173 | 31% |
| **Best** | --- | 282 | 329 | 243 | 255 | 46% |
| **Spread** | --- | 159 | 63 | 107 | 216 | 23% |

**Table S4.** Occurrence of the various score values as observed in all the consensus models generated by using the Glide docking results.

| **Score** | **without spaces** | **isomeric space** | **binding space** | **both spaces merged** | **both spaces joint** | **relative abundance** |
| --- | --- | --- | --- | --- | --- | --- |
| **Primary** | 353 | 385 | 322 | 335 | 382 | 61% |
| **PLANTS** | 42 | 61 | 71 | 59 | 53 | 10% |
| **Xscore** | 12 | 9 | 10 | 49 | 17 | 3% |
| **VEGA** | 65 | 43 | 76 | 93 | 136 | 14% |
| **MLP** | 2 | 44 | 60 | 16 | 30 | 5% |
| **contacts** | 48 | 37 | 43 | 22 | 27 | 6% |
| **APBS** | 0 | 9 | 0 | 0 | 6 | 0% |
| **Type of score values (only for analyses including space descriptors)** | | | | | | |
| **Mean** | --- | 137 | 116 | 163 | 202 | 26% |
| **Best** | --- | 269 | 373 | 256 | 267 | 48% |
| **Spread** | --- | 182 | 93 | 155 | 182 | 26% |

**Table S6.** Occurrence of the various score values as found in all the generated consensus models by all considered docking programs.

| **Score** | **without spaces** | **isomeric space** | **binding space** | **both spaces merged** | **both spaces joint** | **relative abundance** |
| --- | --- | --- | --- | --- | --- | --- |
| **Primary** | 866 | 951 | 775 | 832 | 751 | 36% |
| **PLANTS** | 498 | 552 | 666 | 706 | 754 | 27% |
| **Xscore** | 418 | 450 | 436 | 428 | 330 | 18% |
| **VEGA** | 81 | 111 | 195 | 163 | 390 | 8% |
| **MLP** | 8 | 69 | 81 | 81 | 85 | 3% |
| **contacts** | 203 | 188 | 166 | 125 | 215 | 8% |
| **APBS** | 39 | 33 | 0 | 1 | 35 | 1% |
| **Type of score values** | | | | | | |
| **Mean** | --- | 548 | 515 | 603 | 575 | 24% |
| **Best** | --- | 1021 | 1290 | 1048 | 1018 | 48% |
| **Spread** | --- | 694 | 406 | 563 | 866 | 26% |

**
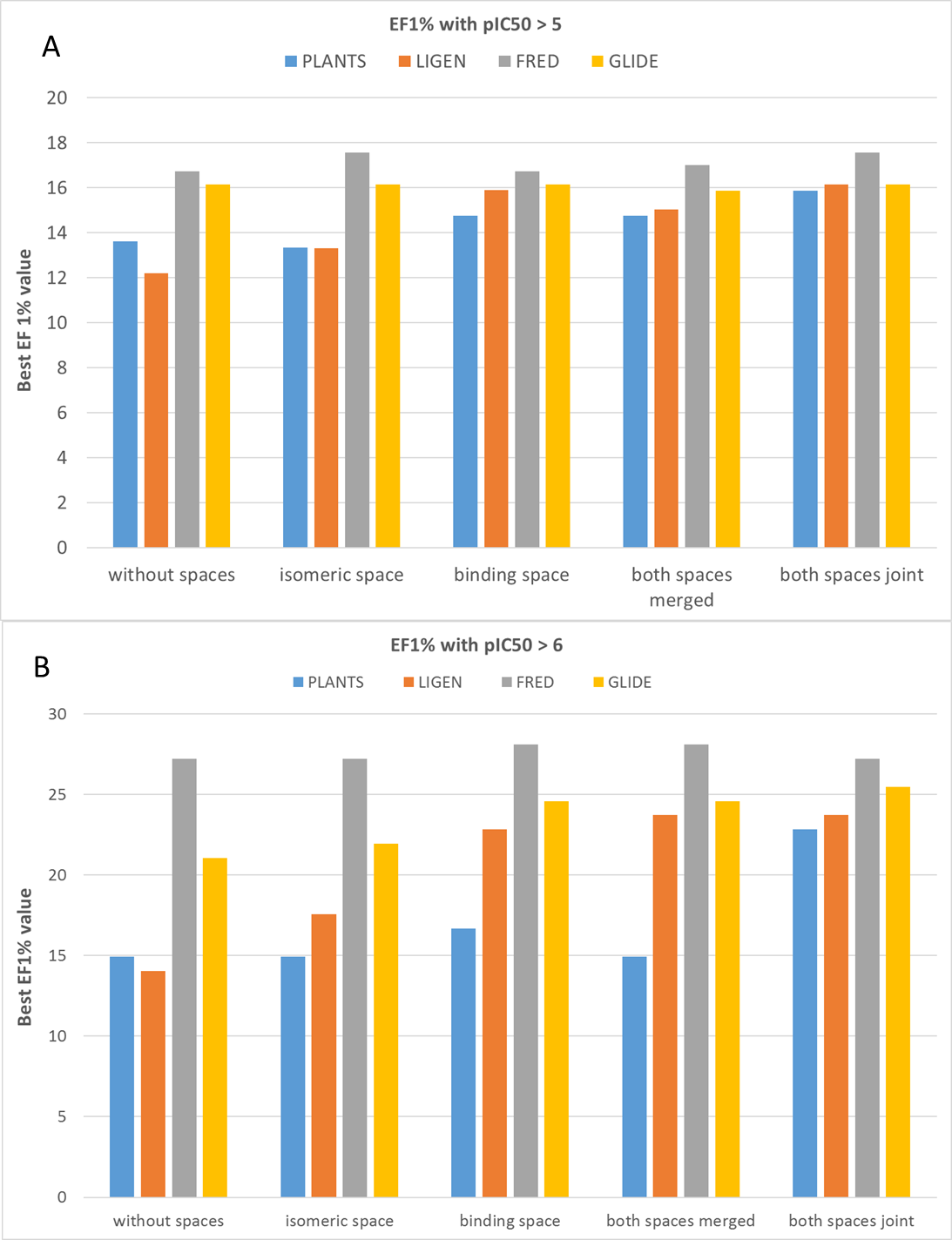
**

**Figure S1.** Best EF1% values as obtained by the four tested docking programs in the five experimented conditions when using the common databases with pIC50 > 5 (1A) and pIC50 > 6 (1B). The analysis of Figure S1 and Table S1 highlights a substantial agreement between the EF1% values computed by using this common database and those obtained by the full databases. The major differences involve the isomeric space, a finding which can be explained by considering that the number of active molecules existing in multiple states is here further reduced, thus minimizing the effect of the isomeric space parameters as exemplified by the almost vanished role played by the isomeric space for the Glide results.


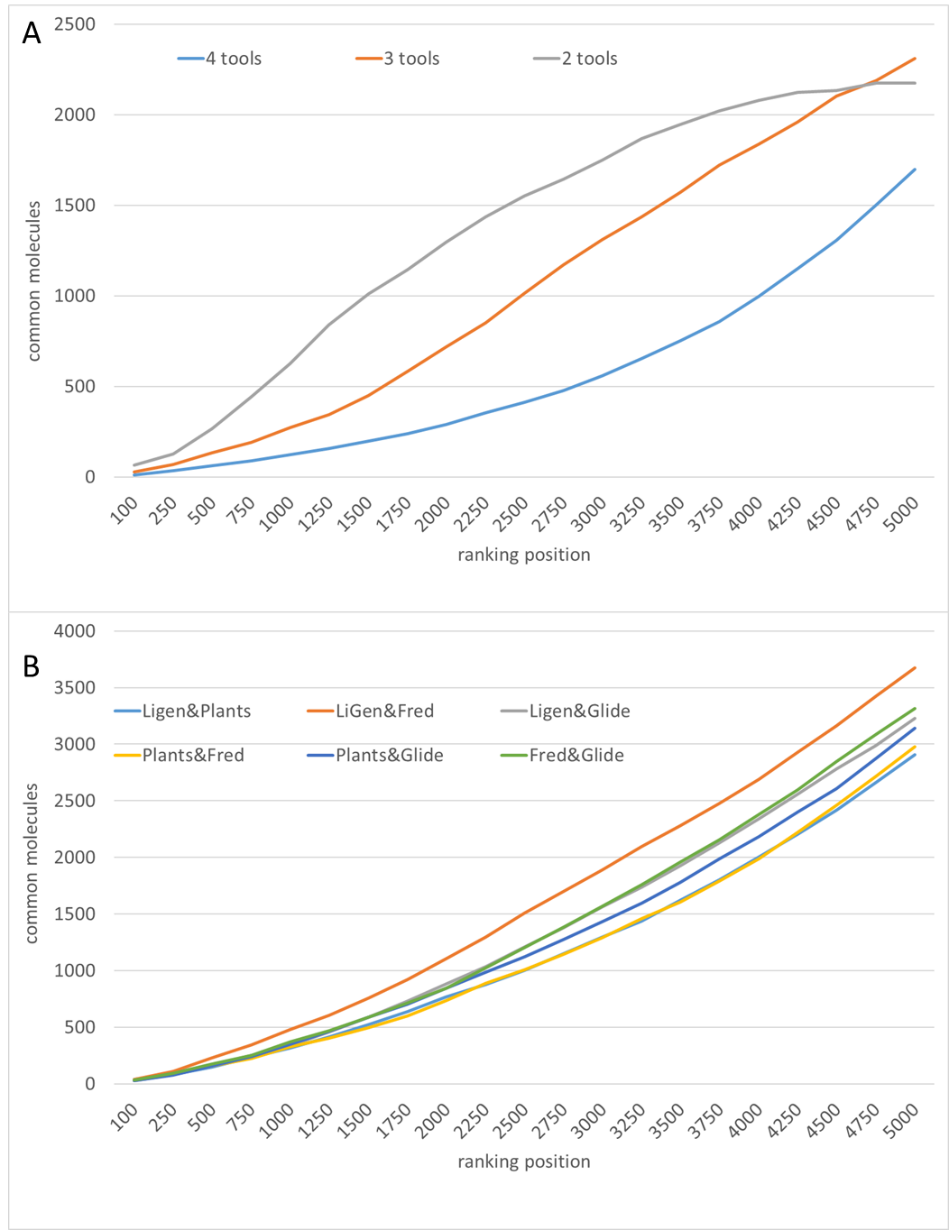


**Figure S2.** Trends of the frequency of the molecules shared at the same time by two, three, or four rankings (S1A) or by specific pairs of ranking (S1B) when browsing the first half of the ranking positions (from 1 to 5000).


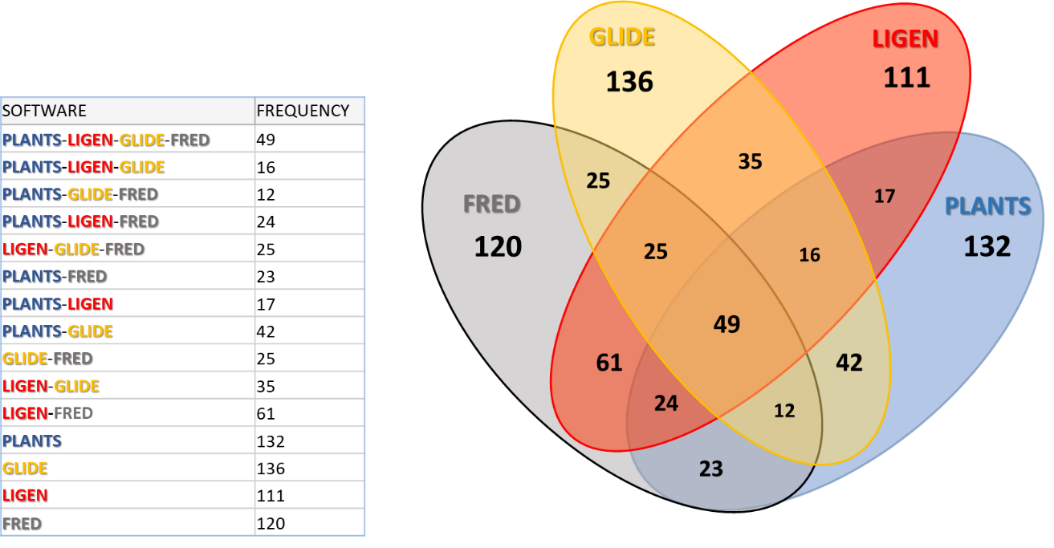


**Figure S3.** Venn diagram showing the frequencies of the scaffolds detected within the Top500 molecules of the four computed rankings (the color code is the same of the Figures 1 and 3 in the main text). The analysis is based on an abstracted scaffold representation, as obtained first identifying all the rings and chains connecting rings of the original molecule and then removing both bond and atom types.


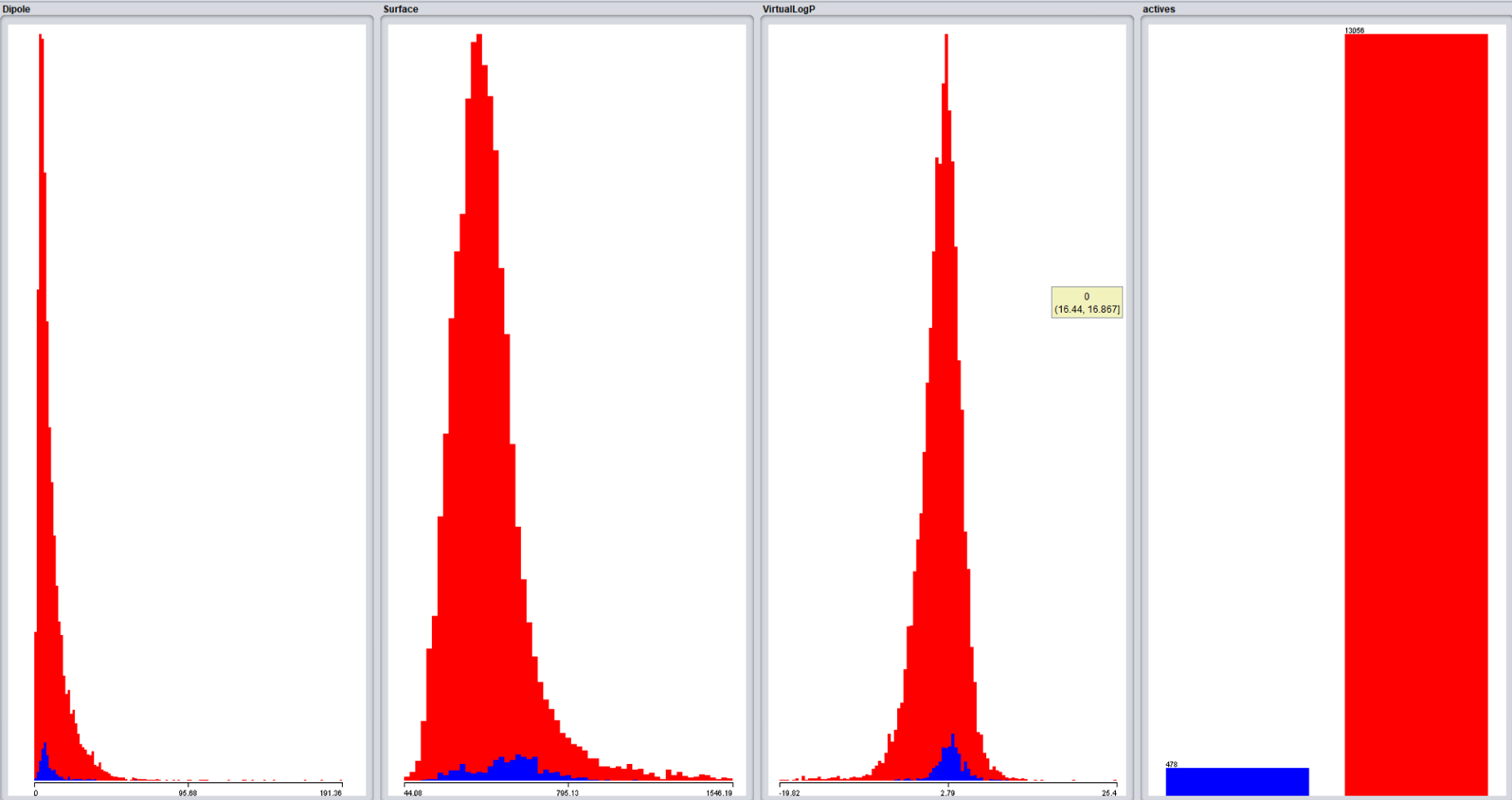


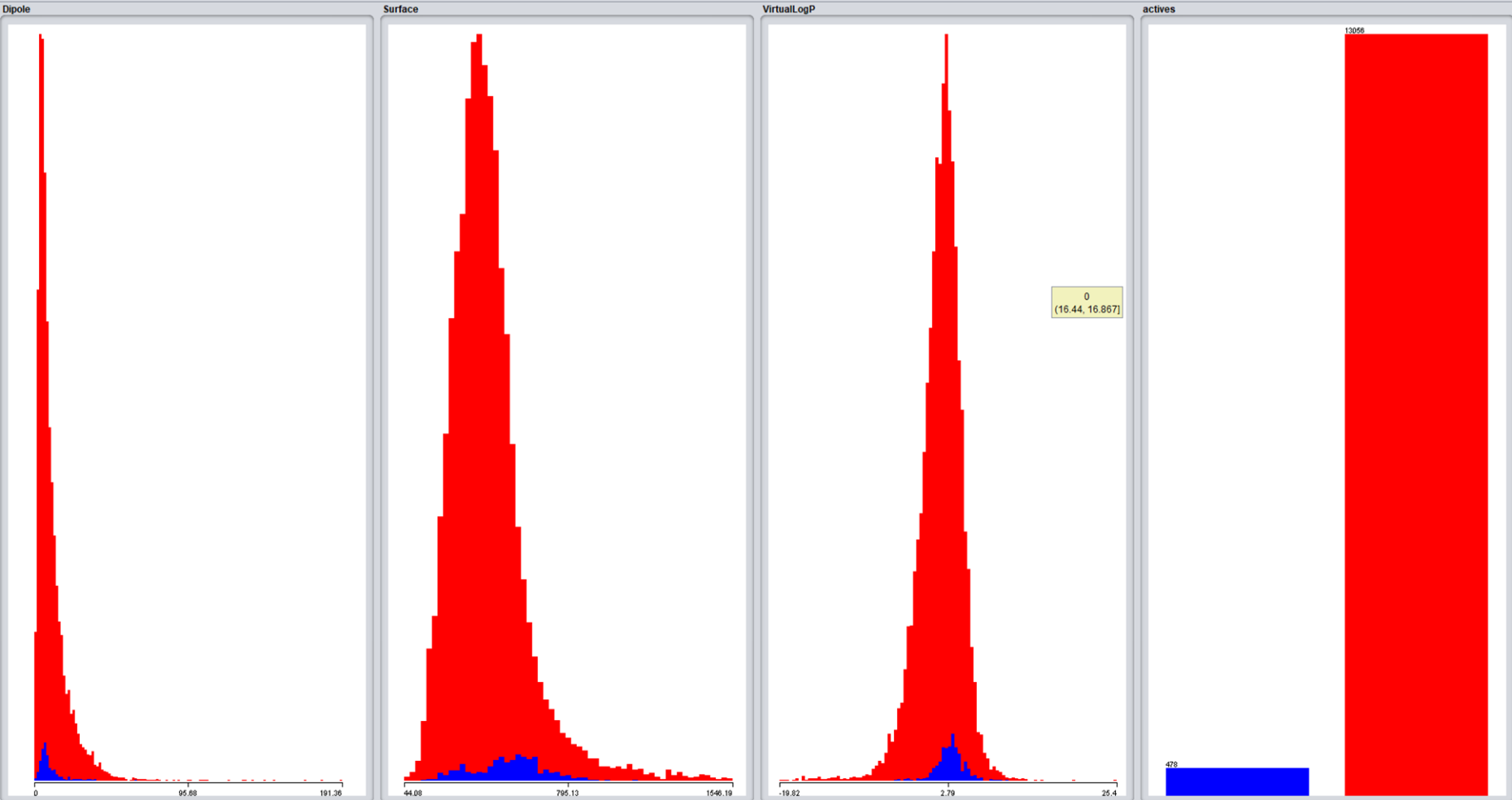


**Figure S4.** Distribution of some representative physicochemical properties between actives (in blue) and inactive molecules (in red).
